# Association of *CYP1B1* L432V polymorphism with urinary cancer susceptibility: a meta-analysis

**DOI:** 10.1186/1746-1596-9-113

**Published:** 2014-06-09

**Authors:** Weifan Jiang, Guang Sun, Jianhua Xiong, Xiaoqing Xi, Zimin Shi

**Affiliations:** 1 Department of Urology, the Second Affiliated Hospital, Nanchang University, Nanchang, China; 2 Department of Urology, The Second Hospital of Tianjin Medical University, Tianjin Institute of Urology, Tianjin, China

**Keywords:** *CYP1B1*, Polymorphism, Urinary cancer, Susceptibility, Meta-analysis

## Abstract

**Abstract:**

**Background**

The Cytochrome P450 1B1 (*CYP1B1*) is a key P450 enzyme involved in the metabolism of exogenous and endogenous substrates. Previous studies have reported the existence of *CYP1B1* L432V missense polymorphism in prostate, bladder and renal cancers. However, the effects of this polymorphism on the risk of these cancers remain conflicting. Therefore, we performed a meta-analysis to assess the association between L432V polymorphism and the susceptibility of urinary cancers.

**Methods**

We searched the PubMed database without limits on language for studies exploring the relationship of *CYP1B1* L432V polymorphism and urinary cancers. Article search was supplemented by screening the references of retrieved studies manually. Odds ratios (OR) and 95% confidence intervals (95% CI) were calculated to evaluate the strength of these associations. Simultaneously, publication bias was estimated by funnel plot and Begg’s test with Stata 11 software.

**Results**

We observed a significant association between *CYP1B1* L432V polymorphism and urinary cancers. The overall OR (95% CI) of CC versus CG was 0.937 (0.881-0.996), the overall OR (95% CI) of CC versus CG + GG was 0.942 (0.890-0.997). Furthermore, we identified reduced risk for CC versus other phenotypes in both prostate and overall urinary cancers, when studies were limited to Caucasian or Asian patients.

**Conclusions**

This meta-analysis suggests that the *CYP1B1* L432V polymorphism is associated with urinary cancer risk.

**Virtual Slides:**

The virtual slide(s) for this article can be found here: http://www.diagnosticpathology.diagnomx.eu/vs/3108829721231527

## Background

Prostate cancer, urothelial carcinoma and renal cancer are common cancer types and major cause of cancer-related death worldwide [1,2]. Smoking, diet and environmental factors have been reported to contribute to the carcinogenesis of these malignancies [3,4]. However, the fact that a small fraction of people exposed to these carcinogens eventually develop urinary cancers suggests that individual genetic predisposition factors may contribute to carcinogenesis.

Cytochrome P450 1B1 (*CYP1B1*) is a member of the CYP1 gene family and one of the major enzymes involved in the hydroxylation of estrogens, involved in the oxidative activation and deactivation of xenobiotics [5-7]. Several polymorphisms in the *CYP1B1* gene have been reported, including 4326C/G (L432V, rs1056836) in exon 3, which encodes the heme-binding domain, have been associated with enhanced catalytic activity when compared to the wild-type allele [8-10].

Polymorphisms in *XPC* and *MHTFR* gene have been reported to be associated with overall urinary cancer risk [11,12], suggesting that urinary cancers share common mechanisms in the process of DNA repair and carcinogen metabolism. Several case–control studies were performed to identify the association of *CYP1B1* polymorphisms with prostate, bladder and renal cancer risk. However, small sample sizes and limited populations in study design have often yielded inclusive results among the studies [13-29]. The inconsistent conclusions may have resulted from difference ethnic backgrounds and relatively small sample sizes. To validate the potential association between the *CYP1B1* Leu432Val polymorphism and urinary cancer risk, we conducted a meta-analysis of data reported in 17 studies including 7,944 cases and 7,389 controls.

## Methods

### Publication search

Medline, PubMed, Embase and Web of Science were searched for all relevant articles with the following terms: “Cytochrome P450 1B1” or “*CYP1B1*”, “polymorphism” or “variant”, “case–control”, “risk”, “association”, “prostate cancer”, “bladder cancer” and “renal cancer” (last search was updated on Feb 10, 2014). References of the retrieved articles on this topic were also manually screened for additional relevant eligible studies.

### Selection criteria

We defined inclusion criteria as follows: written in English; case–control design; sufficient information for estimating odds ratio (OR) and their 95% confidence interval (CI); observed genotype frequencies in the controls in agreement with Hardy-Weinberg equilibrium (HWE). Abstracts and unpublished reports were not considered. Investigations in subjects with family history or cancer-prone disposition were also excluded. Meanwhile, if studies had overlapping subjects, we selected the most recent study that included the largest number of individuals in the publications. This study was approved by the ethics committee of the Second Affiliated Hospital of Nanchang University.

### Data extraction

Two investigators independently extracted the following information from each study: the first author, year of publication, country of origin, ethnicity, source of controls (population-based, hospital-based and mixed controls), genotyping method, number of genotyped cases and controls, number of genotypes for three *CYP1B1* polymorphisms in cases and controls, and main findings.

### Statistical methods

Hardy–Weinberg equilibrium (HWE) was evaluated for each study, using the goodness-of-fit chi-square test. *P* < 0.05 was considered representative of departure from HWE. Crude OR with 95% CI was used to assess the strength of association between the three *CYP1B1* L432V polymorphism and urinary cancer risk. Then, we calculated the pooled ORs and 95% CIs. Heterogeneity assumption was checked by the chi-square-based Q-test. A *P* value greater than 0.10 for the Q-test indicates a lack of heterogeneity among studies, so the pooled OR estimate of the each study was calculated by the fixed-effects model (the Mantel–Haenszel method), the random-effects model (the Der-Simonian and Laird method) was used otherwise [30,31]. To assess the effects of individual studies on the overall risk of cancer, sensitivity analysis was performed by excluding each study at a time individually and recalculating the ORs and 95% CIs. We also used the inverted funnel plot and the Egger’s test to examine the potential influence of publication bias (linear regression analysis). The significance of the intercept was determined by the t-test suggested by Egger (*P* < 0.05 was considered representative of statistically significant publication bias) [32]. All statistical tests were two-sided, and a P-value of < 0.05 was considered statistically significant. Statistical tests were performed with STATA version 11.0 (Stata Corporation, College Station, TX) or SAS software (version 9.1; SAS Institute, Cary, NC).

## Results and discussion

### Study characteristics

**Figure 1:**
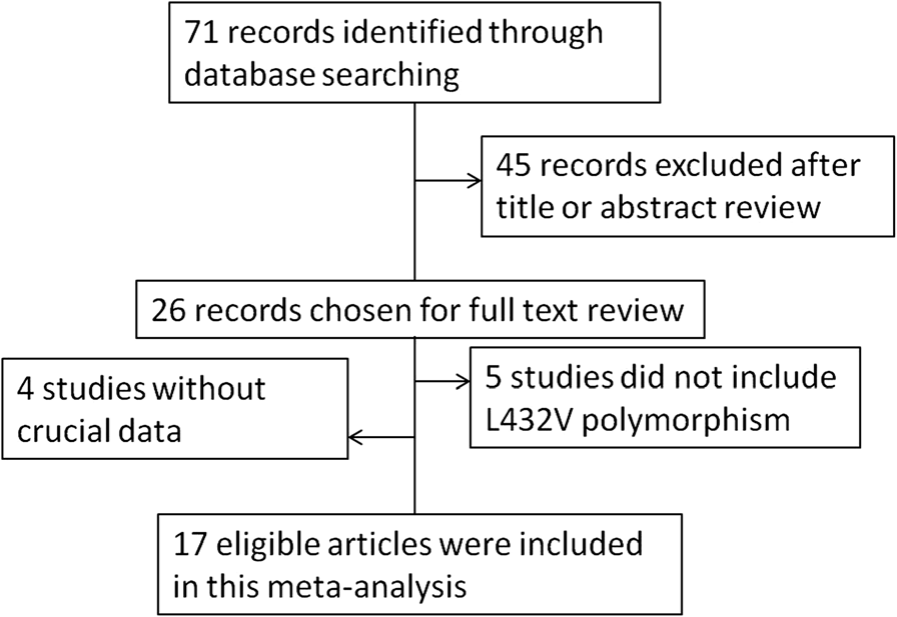
The flow chart of the included studies in the meta-analysis.

We identified a total of 71 relevant publications after initial screening. Among these, 26 publications had met the inclusion criteria and were subjected to further examination. We excluded 4 publications because they did not present detailed genotyping information. We also excluded 5 publications because they did not include L432V polymorphism. Our final data consisted of 10 publications with a total of 5949 cases and 5388 controls for prostate cancer, 5 publications with 1658 cases and 1593 controls for bladder cancer, 2 publications with 337 cases and 408 controls for renal cancer (Figure 1). Of these, there were 10 hospital-based studies and 7 population-based studies. Characteristics of included studies are summarized in Table 1.

**Table 1:** **Characteristics of studies included in the meta-analysis for an association between *CYP1B1 *** **L432V SNP and risk of urinary cancer**

**First author, year**	**Country**	**Ethnicity**	**Source of control**	**Cases/controls**	**MAF of controls**	**Power**^**a**^	**Source of DNA**	**Genotyping methods**
**Prostate cancer**								
Holt, 2013	USA	Caucasian	PB	1256/1235	0.41	1	blood	SNPlex
Catsburg C, 2012	USA	Caucasian	PB	1433/760	0.47	1	blood	Taqman
Beuten J, 2008	USA	Caucasian	PB	649/738	0.39	1	blood	Taqman
Berndt, 2007	USA	Mixed	PB	486/611	0.47	1	blood	TaqMan
Cussenot O, 2007	France	Caucasian	HB	1053/837	0.39	1	blood	TaqMan
Sobti RC, 2006	India	Asian	PB	100/100	0.18	0.415	blood	PCR–RFLP
Cicek MS, 2005	USA	Mixed	HB	439/479	0.47	1	blood	PCR–RFLP
Fukatsu, 2004	Japan	Asian	HB	136/255	0.29	0.947	blood	PCR–RFLP
Chang BL, 2003	USA	Mixed	HB	310/182	0.44	0.807	ND	Sequencing
Tanaka Y, 2002	Japan	Asian	PB	117/200	0.18	0.857	ND	AS-PCR
**Bladder cancer**								
Berber U, 2013	Turkey	Asian	PB	114/114	0.27	0.499	tissue	AS-PCR
Salinas-Sánchez AS, 2012	Spain	Caucasian	HB	208/208	0.39	0.875	blood	Sequencing
Fontana L, 2009	France	Caucasian	HB	51/45	0.58	0.104	blood	TaqMan
Figueroa J, 2008	Spain	Caucasian	HB	1084/1012	0.42	1	blood/buccal	TaqMan
Hung RJ, 2004	Italy	Caucasian	HB	201/214	0.59	0.888	ND	PCR–RFLP
**Renal cancer**								
Salinas-Sánchez AS, 2012	Spain	Caucasian	HB	126/208	0.39	0.875	blood	Sequencing
Sasaki M, 2005	USA	Asian	HB	211/200	0.18	0.857	tissue	AS-PCR

Abbreviations: *SNP* Single nucleotide polymorphism, *HB* Hospital based, *PB* Population based, *RFLP* Restriction fragment length polymorphisms, *AS-PCR* Allele specific PCR, *ND* Not described. ^a^Statistical power to detect an OR of 1.5 (or 0.67 = 1/1.5).

### Quantitative synthesis

**Figure 2:**
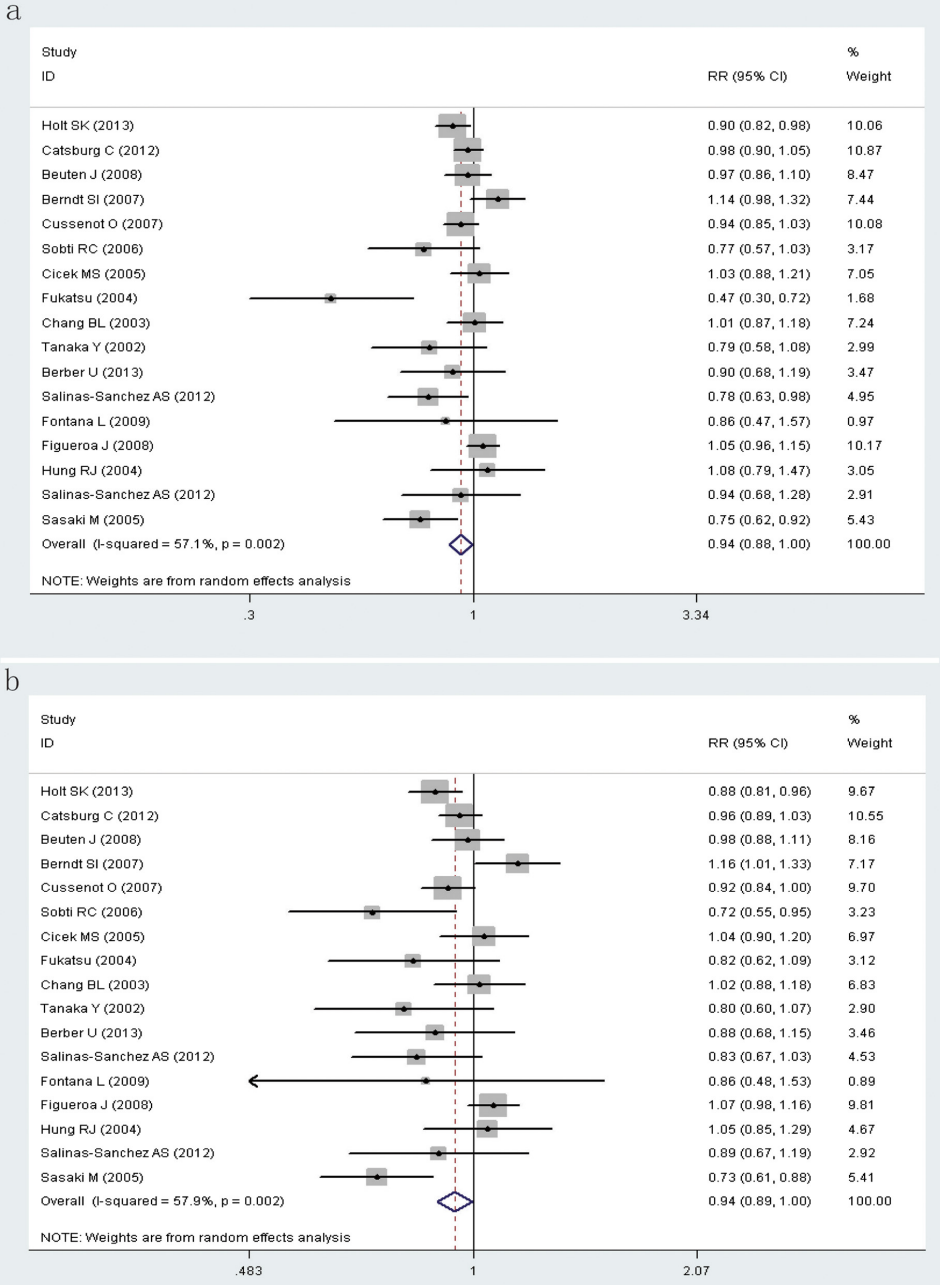
**Forest plot (Random effects model) describing the association of the *CYP1B1 *L432V polymorphism with risk of urinary cancers.** The CC phenotype was associated with a modestly decreased risk of urinary cancers (**a**, CC vs CG; **b**, CC vs CG + GG).

Table 2 lists the main results of this meta-analysis. Overall, significant associations were found between *CYP1B1* L432V polymorphism and urinary cancer risk when all studies pooled into the meta-analysis (CC vs CG: OR = 0.937, 95% CI = 0.881-0.996; CC vs CG + GG: OR = 0.942, 95% CI = 0.890-0.997; C vs G: OR = 0.957, 95% CI = 0.917-0.998) (Figure 2). In the subgroup analysis, L432V polymorphism was significantly associated with prostate cancer or overall urinary cancer risk when population was defined as only Caucasians or Asians (Additional file 1: Table S1). Nevertheless, when studies were restricted to population-based or hospital-based studies, none of these comparisons showed significant differences. The FPRP values for significant findings at different prior probability levels were also calculated. For a prior probability of 0.1, assuming that the OR for specific genotype was 0.67/1.50 (protection/risk), the FPRP values were 0.786, 0.79 and 0.794 for an association of overall CC vs CG, CC vs CG + GG and C vs G genotypes with an increased lung cancer risk.

**Table 2:** **Meta-analysis of the associations between *CYP1B1 *** **L432V polymorphism and urinary cancer risk**

**Cancer**	**Number of studies**	**Cases/controls**	**Comparison**	**Test of association**			**Test of heterogeneity**			**Publication bias**
				**OR**	**95% CI**	***P * Value**	**Q**	***P *** **Value**	**I**^**2**^**(%)**	***P *** **Value (Begg’s)**
Prostate	10	5949/5388	CC vs GG	0.942	0.868-1.022	0.151	20.23	0.017	55.5	0.917
			CC vs CG	0.947	0.881-1.018	0.137	23.13	0.006	61.1	0.212
			CC vs CG + GG	0.952	0.895-1.013	0.120	20.00	0.018	55.0	0.671
			C vs G	0.963	0.920-1.008	0.108	24.36	0.004	63.0	0.723
Bladder	5	1658/1593	CC vs GG	1.061	0.962-1.171	0.234	2.23	1	0.0	0.035
			CC vs CG	1.000	0.926-1.079	0.991	7.20	0.126	44.4	0.317
			CC vs CG + GG	1.016	0.947-1.090	0.661	6.25	0.181	36.0	0.156
			C vs G	1.026	0.977-1.077	0.312	3.75	0	0.0	0.060
Renal	2	337/408	CC vs GG	0.740	0.593-0.922	0.007	0.73	0.394	0	-
			CC vs CG	0.816	0.686-0.969	0.021	1.34	0.248	25.2	-
			CC vs CG + GG	0.787	0.673-0.922	0.003	1.35	0.245	26.0	-
			C vs G	0.818	0.729-0.917	0.001	1.82	0.178	45	-
Overall	17	7944/7389	CC vs GG	0.941	0.876-1.012	0.103	35.2	0.004	54.6	0.607
			CC vs CG	0.937	0.881-0.996	0.037	37.29	0.002	57.1	0.076
			CC vs CG + GG	0.942	0.890-0.997	0.038	37.97	0.002	57.9	0.199
			C vs G	0.957	0.917-0.998	0.039	46.07	0	65.3	0.302

### Heterogeneity and sensitivity analyses

Heterogeneities were observed among studies for the association between the *CYP1B1* L432V polymorphism and urinary cancer risk (CC vs GG, CC vs CG, CC vs CG + GG, C vs G for prostate cancer; C vs G for bladder cancer; CC vs GG, CC vs CG, CC vs CG + GG, C vs G for overall urinary cancers). Therefore, we used the random-effects model that generated wider CIs. For the other groups of comparisons, no heterogeneity was found among studies and the fixed-effects model was performed (Table 2, Additional file 1: Table S1). The leave-one-out sensitivity analysis indicated that no single study changed the pooled ORs qualitatively (data not shown).

### Publication bias

The shapes of the funnel plots seemed symmetrical, and Egger’s test suggested that publication bias was only found in the CC vs GG group of bladder cancer (Table 2, Additional file 1: Table S1).

In this meta-analysis, we tested the association between L432V polymorphism in the *CYP1B1* gene and urinary cancer risk by comparing the allele frequencies from 17 published studies. We observed a significant association of L432V polymorphism with overall urinary cancer risk, as well as in the subgroups defined as Caucasian or Asian populations. Urinary system tumorigenesis is a complex event, in which different carcinogenic chemicals are involved. Prostate is a hormone-responsive organ in which androgens are believed to stimulate growth and secretory functions. Evidences have been shown that *CYP1B1* protein is highly expressed in prostate cancer tissues, while not in normal prostate tissues [33]. It has been reported that different allelic variants of *CYP1B1* have different catalytic activities and specificities to procarcinogens, thus partly explains molecular mechanism of *CYP1B1* in carcinogenesis [34]. Except for prostate cancer, case–control studies have shown inconsistent associations between *CYP1B1* L432V polymorphism and bladder cancer and renal cancer risk [24,28,29]. However, exact mechanisms of how *CYP1B1* polymorphism contributes to urinary cancer susceptibility requires further illustration.

Some limitations of this meta-analysis should be discussed. First, the total number of included studies of bladder cancer and renal cancer was relatively small. Second, our results were based on unadjusted estimates, while a more precise analysis is needed if individual data were available, which would allow for the adjustment by other factors such as age, smoking status, drinking status. Finally, unpublished data may have not been included in the current analysis, potentially causing a bias in the results.

## Conclusions

In conclusion, this meta-analysis suggests that the *CYP1B1* L432V polymorphism is associated with urinary cancer development, especially in specified Caucasian and Asian populations. However, studies with larger number of samples from homogeneous urinary cancer patients are needed. Further biological investigations may eventually lead to better understanding of the association between the *CYP1B1* polymorphism and urinary cancer risk.

## Competing interests

The authors declare that they have no competing interests.

## Authors’ contributions

WF J, G S and JH X conceived and performed statistics, WF J, XQ X and ZM S extracted data and wrote the manuscript, G S and ZM S revised the manuscript. All authors read and approved the final manuscript.

## Supplementary Material

Additional file 1: Table S1 Subgroup analysis of association between *CYP1B1* L432V polymorphism and urinary cancer risk. Click here for file
