# Supplementary material for: Association of CYP1B1 L432V polymorphism with urinary cancer susceptibility: a meta-analysis
Source: Diagn Pathol. 2014 Jun 9;9:113. doi: 10.1186/1746-1596-9-113 (PMC4067118; doi:10.1186/1746-1596-9-113)
Supplement: Additional file 1: Table S1 — Subgroup analysis of association between CYP1B1 L432V polymorphism and urinary cancer risk. [file 1746-1596-9-113-S1.docx]

| **Supplementary Table.**Subgroup analysis of association between *CYP1B1* L432V polymorphism and urinary cancer risk | | | | | | | | | | |
| --- | --- | --- | --- | --- | --- | --- | --- | --- | --- | --- |
| **Cancer** | **Number of Studies** | **Subgroup** | **Comparison** | **Test of association** | | | **Test of heterogeneity** | | | **Publication bias** |
|  |  |  |  | OR | 95% CI | *P* Value | Q | *P* Value | I^2^(%) | P Value (Begg's) |
| Prostate | 4 | Caucasian | CC vs GG | 0.908 | 0.860-0.960 | 0.001 | 3.88 | 0.275 | 22.7 | 0.752 |
|  |  |  | CC vs CG | 0.941 | 0.899-0.986 | 0.011 | 2.15 | 0.542 | 0.0 | 0.869 |
|  |  |  | CC vs CG+GG | 0.931 | 0.891-0.972 | 0.001 | 3.00 | 0.391 | 0.1 | 0.924 |
|  |  |  | C vs G | 0.949 | 0.923-0.976 | 0.000 | 3.56 | 0.313 | 15.7 | 0.661 |
| Prostate | 3 | Asian | CC vs GG | 0.810 | 0.651-1.008 | 0.059 | 3.12 | 0.210 | 35.9 | 0.933 |
|  |  |  | CC vs CG | 0.740 | 0.606-0.904 | 0.003 | 4.47 | 0.107 | 55.2 | 0.103 |
|  |  |  | CC vs CG+GG | 0.784 | 0.666-0.922 | 0.003 | 0.51 | 0.775 | 0.0 | 0.589 |
|  |  |  | C vs G | 0.821 | 0.726-0.928 | 0.002 | 1.51 | 0.469 | 0.0 | 0.757 |
| Prostate | 6 | PB | CC vs GG | 0.926 | 0.815-1.052 | 0.239 | 16.29 | 0.006 | 69.3 | 0.841 |
|  |  |  | CC vs CG | 0.953 | 0.876-1.037 | 0.262 | 11.24 | 0.047 | 55.5 | 0.578 |
|  |  |  | CC vs CG+GG | 0.941 | 0.856-1.034 | 0.205 | 16.12 | 0.007 | 69.0 | 0.645 |
|  |  |  | C vs G | 0.952 | 0.888-1.020 | 0.163 | 19.34 | 0.002 | 74.1 | 0.651 |
| Prostate | 4 | HB | CC vs GG | 0.943 | 0.865-1.027 | 0.178 | 3.88 | 0.274 | 22.7 | 0.497 |
|  |  |  | CC vs CG | 0.916 | 0.777-1.080 | 0.295 | 11.93 | 0.008 | 74.9 | 0.381 |
|  |  |  | CC vs CG+GG | 0.953 | 0.893-1.017 | 0.146 | 3.84 | 0.280 | 21.8 | 0.949 |
|  |  |  | C vs G | 0.966 | 0.925-1.009 | 0.119 | 4.92 | 0.177 | 39.1 | 0.878 |
| Bladder | 4 | Caucasian/HB | CC vs GG | 1.075 | 0.972-1.190 | 0.159 | 0.87 | 0.833 | 0.0 | 0.029 |
|  |  |  | CC vs CG | 1.008 | 0.931-1.092 | 0.838 | 6.55 | 0.088 | 54.2 | 0.489 |
|  |  |  | CC vs CG+GG | 1.027 | 0.955-1.105 | 0.469 | 4.99 | 0.173 | 39.8 | 0.326 |
|  |  |  | C vs G | 1.034 | 0.983-1.088 | 0.193 | 1.93 | 0.587 | 0.0 | 0.159 |
| Overall | 9 | Caucasian | CC vs GG | 0.946 | 0.902-0.993 | 0.024 | 13.71 | 0.090 | 41.7 | 0.746 |
|  |  |  | CC vs CG | 0.958 | 0.921-0.997 | 0.035 | 10.86 | 0.210 | 26.4 | 0.573 |
|  |  |  | CC vs CG+GG | 0.955 | 0.920-0.991 | 0.014 | 13.54 | 0.095 | 40.9 | 0.672 |
|  |  |  | C vs G | 0.968 | 0.945-0.992 | 0.010 | 14.58 | 0.068 | 45.1 | 0.864 |
| Overall | 5 | Asian | CC vs GG | 0.780 | 0.667-0.913 | 0.002 | 4.19 | 0.381 | 4.5 | 0.686 |
|  |  |  | CC vs CG | 0.776 | 0.683-0.880 | 0.000 | 6.28 | 0.179 | 36.3 | 0.352 |
|  |  |  | CC vs CG+GG | 0.783 | 0.701-0.875 | 0.000 | 1.88 | 0.758 | 0.0 | 0.351 |
|  |  |  | C vs G | 0.817 | 0.751-0.888 | 0.000 | 3.41 | 0.491 | 0.0 | 0.361 |
| Overall | 7 | PB | CC vs GG | 0.922 | 0.819-1.037 | 0.175 | 16.49 | 0.011 | 63.6 | 0.756 |
|  |  |  | CC vs CG | 0.954 | 0.908-1.001 | 0.055 | 11.44 | 0.076 | 47.5 | 0.492 |
|  |  |  | CC vs CG+GG | 0.937 | 0.859-1.022 | 0.144 | 16.4 | 0.012 | 63.4 | 0.556 |
|  |  |  | C vs G | 0.948 | 0.889-1.012 | 0.109 | 19.72 | 0.003 | 69.1 | 0.554 |
| Overall | 10 | HB | CC vs GG | 0.955 | 0.866-1.053 | 0.358 | 17.59 | 0.040 | 48.8 | 0.481 |
|  |  |  | CC vs CG | 0.918 | 0.830-1.015 | 0.095 | 25.86 | 0.002 | 65.2 | 0.117 |
|  |  |  | CC vs CG+GG | 0.942 | 0.869-1.022 | 0.152 | 21.11 | 0.012 | 57.4 | 0.221 |
|  |  |  | C vs G | 0.961 | 0.904-1.020 | 0.192 | 25.34 | 0.003 | 64.5 | 0.307 |
